# Malaria and tuberculosis as diseases of neglected populations: state of the art in chemotherapy and advances in the search for new drugs

**DOI:** 10.1590/0074-02760200229

**Published:** 2020-09-30

**Authors:** Renan Vinicius de Araújo, Soraya Silva Santos, Luccas Missfeldt Sanches, Jeanine Giarolla, Omar El Seoud, Elizabeth Igne Ferreira

**Affiliations:** 1 Universidade de São Paulo, Faculdade de Ciências Farmacêuticas, Departamento de Farmácia, Laboratório de Planejamento e Síntese de Quimioterápicos Contra Doenças Negligenciadas, São Paulo, SP, Brasil; 2 Universidade de São Paulo, Instituto de Química, Departamento de Química Fundamental, São Paulo, SP, Brasil

**Keywords:** *Plasmodium* sp., Mycobacterium tuberculosis, neglected populations chemotherapy, new drugs

## Abstract

Malaria and tuberculosis are no longer considered to be neglected diseases by the World Health Organization. However, both are huge challenges and public health problems in the world, which affect poor people, today referred to as neglected populations. In addition, malaria and tuberculosis present the same difficulties regarding the treatment, such as toxicity and the microbial resistance. The increase of *Plasmodium* resistance to the available drugs along with the insurgence of multidrug- and particularly tuberculosis drug-resistant strains are enough to justify efforts towards the development of novel medicines for both diseases. This literature review provides an overview of the state of the art of antimalarial and antituberculosis chemotherapies, emphasising novel drugs introduced in the pharmaceutical market and the advances in research of new candidates for these diseases, and including some aspects of their mechanism/sites of action.

Malaria (Fig. 1) is one of the most prevalent parasitic diseases worldwide, present in 89 countries around the world.^1^ ^,^ ^2^ This illness is an acute febrile infectious disease, whose etiological agent is a protozoan of the genus *Plasmodium*. It is estimated that there were 228 million cases and 405,000 deaths in 2019, within a population of over 3 billion at risk of infection.^1^ ^,^ ^3^ This scenario as well as *Plasmodium* resistance to the available drugs demonstrate the urgent need for developing new therapeutic options.^1^ The disease is transmitted mainly through the bite of the *Anopheles* mosquito and five *Plasmodium* species infect human beings: *P. vivax*, *P. falciparum*, *P. malariae*, *P. ovale* and *P. knowlesi*. The latter is mostly found in monkeys and, incidentally, can infect people. Furthermore, *P. falciparum* infections account for most deaths, whilst *P. vivax* infections may induce severe chronic malaria.^4^ ^,^ ^5^

**Fig. 1:**
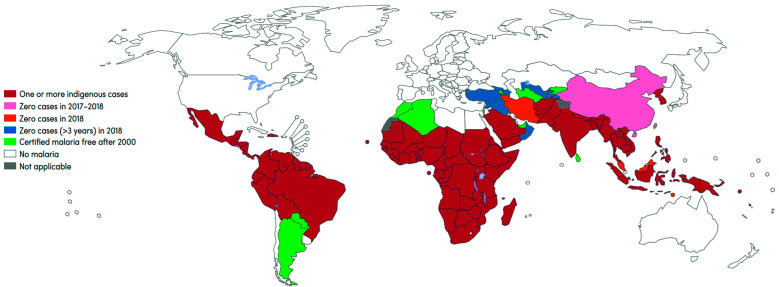
indigenous cases of malaria status, 2019.^1^

Tuberculosis (TB) is an infectious disease caused by *Mycobacterium tuberculosis* (*M. tuberculosis*) and is still one of the greatest problems for public health around the world today. It affects mainly the lungs; however, the brain, liver and other organ systems can be affected as well. In 2018, approximately 10.0 million people fell ill and 1.4 million died of TB, according to the World Health Organization (WHO) Global tuberculosis report released in 2019, as seen in Fig. 2. The WHO estimates around one third of the total human population might be infected with the TB latent form. The insurgence of multidrug-(MDR-TB) and extensively drug-resistant (XDR-TB) strains is alarming and increases the severity and difficulty of the treatment.^6^ ^,^ ^7^

Both diseases are huge challenges and public health problems in the world, which affect the same populations, generally poor people. Furthermore, malaria and tuberculosis present the same troubles regarding the difficulty in treatment, drug toxicity and the microbial resistance to the available medicines. This scenario is enough to justify efforts towards the development of novel therapeutic agents.

**Fig. 2:**
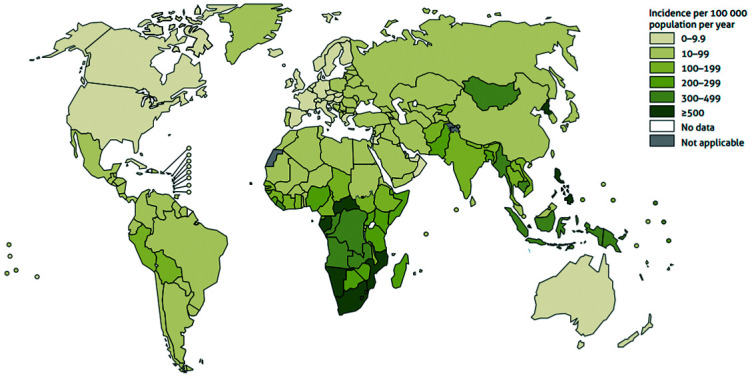
estimated tuberculosis incidence rates, 2018.^6^

Malaria chemotherapy

*Current status* - The treatment regimen for malaria depends heavily on several parameters, such as age, pregnancy, species, severity and chronicity. For this reason, there is a wide array of drugs employed and there are different drug regimens possible, making malaria treatment heterogeneous and diverse. The main drugs employed for malaria therapy are: artemisinins, endoperoxides and derivatives (mainly artesunate, artemether, dihydroartemisinin), primaquine, due to its gametocytocidal effect and its activity against the hypnozoite form (present only in *P. vivax* and *P. ovale*), quinine and 4-aminoquinoline derivatives, such as chloroquine and amodiaquine, and the antifolate agents sulfadoxine/pyrimethamine, used in combination. The WHO recommends artemisinin-based combination therapies (ACTs) for the treatment of uncomplicated *P. falciparum* and *P. vivax* malaria,^8^ which has led to its widespread adoption as a first-line therapy.^9^ ACTs combine an artemisinin derivative with other drugs with different mechanisms of action. However, parasite strains resistant to the drugs currently in use have emerged, especially for chloroquine, whose widespread utilisation causes resistance in most of the endemic regions.^5^ ^,^ ^10^ ^,^ ^11^

Malaria chemotherapy has multiple biochemical pathways and enzymes as targets, some of which are still unknown. Current chemotherapy explores mainly the formation of hemozoin crystals,^12^ ^,^ ^13^ oxidative stress via generation of reactive oxygen species (ROS),^14^ parasitic protein kinases,^15^ and the folic acid biosynthesis pathway.^16^ Some notable new drug targets under investigation are discussed below.

*Advances in the research of new antimalarial agents* - Artemisinin and its analogs are endoperoxides, whose mechanism of action induces oxidative stress inflicted through ROS formation. The generation of the toxic free radical is dependent on the interaction of the drug with intraparasitic heme groups, present on the food vacuoles of the parasite. It is proposed that, upon activation, the toxic radical promotes the alkylation of several protein targets, which leads to biological function impairment and parasite’s death.^17^ All artemisinin derivatives currently in clinical use are considered equally effective and safe.^9^ Arterolane maleate (AM - from Sun Pharma^®^) is a synthetic 1,2,4-trioxolane with a peroxidic pharmacophore, which exhibited *in vitro* potency higher than most current chemotherapeutic agents against *P. falciparum*, including against chloroquine resistant strains.^18^ ^,^ ^19^ A phase III clinical trial with pediatric patients compared ACT of AM combined with piperaquine phosphate (PQP) against artemether-lumefantrine (AL). The AM-PQP treatment demonstrated efficacy comparable to the current treatment of AL, and a similar safety profile. Currently, it is marketed in India and in several African countries under the brand name Synriam.^20^ ^,^ ^21^ Artefenomel (OZ439) proved to be effective in a phase IIa clinical trial in doses below 200 mg, providing rapid reduction of parasitaemia and symptoms, presenting a good tolerance in doses up to 1600 mg. On account of its long half-life, it is anticipated that it could be administered in a single oral dose. Nowadays, the drug is in a phase IIb clinical trial in combination with ferroquine, with plans to have it administered in combined therapy with piperaquine and DSM265.^22^ ^,^ ^23^ The main artemisinins are shown in Fig. 3. There are at least 30 artemisinin derivatives (between new molecules and hybrid compounds) in development in several countries around the world.^24^

**Fig. 3:**
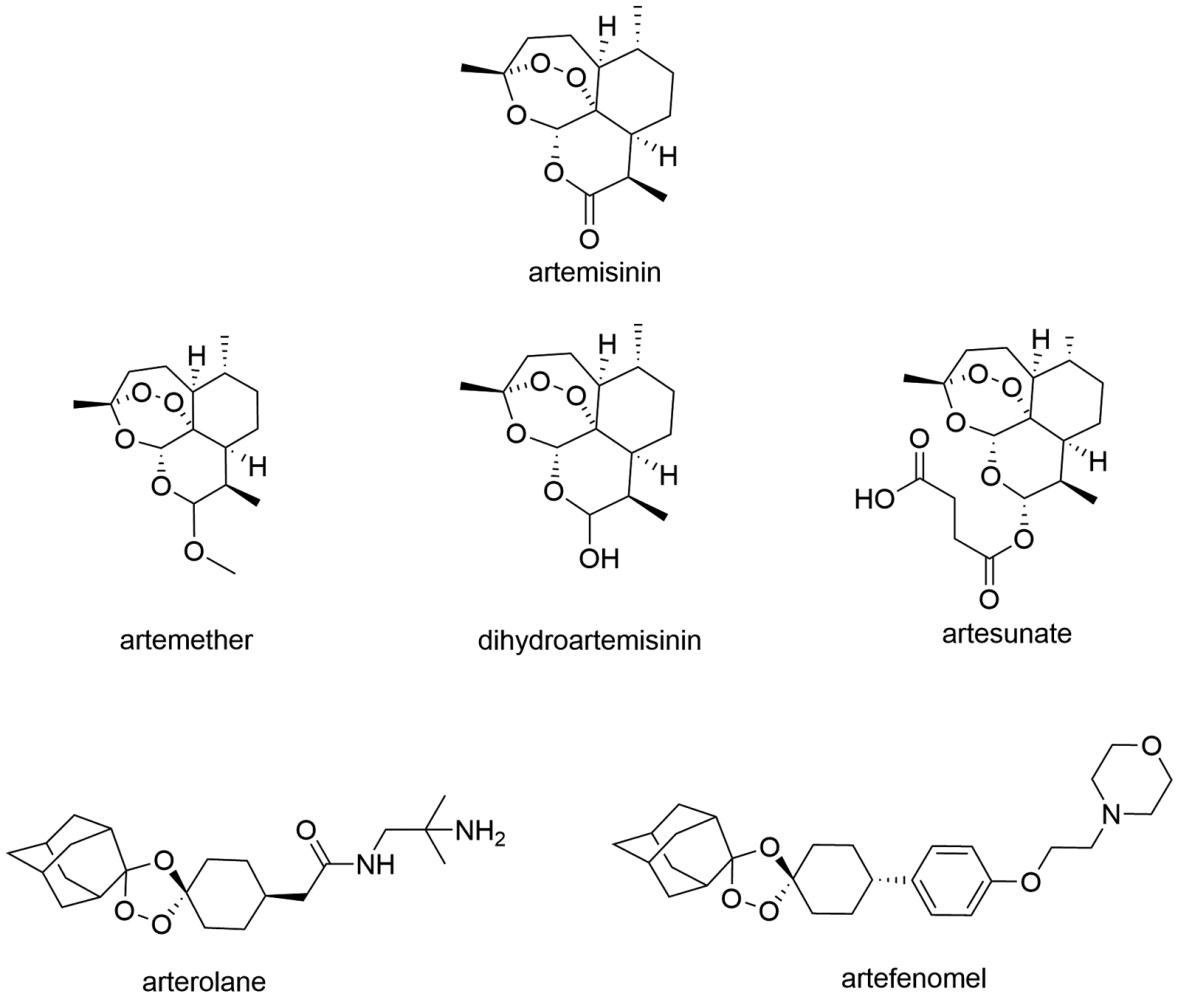
artemisinin and its derivatives.

Several new combination therapy regimens for artemisinins and its derivatives are going through clinical trials in order to establish the most efficient and safe therapeutic scheme, while also monitoring the development of local resistant strains. The ACT artesunate-amodiaquine (ASAQ) and AL regimens were tested in a 42 day trial in the Ivory Coast, and the study found successful treatment rates of 100% and 99.1% respectively, demonstrating that both ACTs are suitable for further use and no resistance is yet identified in the country.^25^ In Ethiopia, the efficacy of treatment with AL was assessed in comparison to chloroquine (CQ), with and without primaquine, as a combined therapy for *P. vivax*. The findings demonstrated that, even though there are signs of CQ-resistant strains in Ethiopia, CQ treatment still exhibited smaller recurrence rates 28 days and 42 days after starting the treatment. Nevertheless, both treatment regimens were improved when co-administered with PQ, evidencing that the treatment schedule would further benefit patients at risk of relapsed infection and transmission.^26^ A similar study was performed in the Brazilian Amazon, assessing the efficacy of ASAQ against CQ. In this report, ASAQ presented a higher efficacy, displaying a faster clearance of fever and parasitaemia. Moreover, the study estimated the expected CQ resistance prevalence within the region to be around 11%.^27^ In Thailand, the pharmacokinetics/pharmacodynamics and electrocardiographic effects of dihydroartemisinin-piperaquine (DHAP) combination therapy were evaluated. These results indicate that DHAP would be unlikely to induce a prolongation of the QT interval over 50 milliseconds, hence the negligible association with this type of cardiac complication.^28^ Another report, carried out by Chhonker et al., investigated the drug-drug interactions between intramuscular α/β-arteether and oral sulfadoxine-pyrimethamine. Herein, both treatments were found not to interfere significantly with each other’s pharmacokinetics in the reported group of healthy volunteers, supporting the use of these two treatments as a new combination therapy for malaria.^29^

Naphthoquine (Fig. 4) is a 4-aminoquinoline developed in China and synthesised in 1986. Its discovery was followed by clinical trials and its combination with artemisinin was approved in 2003 by the China Food and Drug Administration. Although it is considered safe and efficacious, the manufacturing company did not meet the WHO ´s manufacturing standards and single dose use is not in accordance with the 3-day regimen guideline for ACTs.^30^ A clinical trial conducted in China evaluated efficacy and safety of a 3-day treatment of artemisinin/naphthoquine as compared to chloroquine/primaquine. Artemisinin/naphthoquine was shown to be efficacious and safe.^31^ A phase III trial conducted in Indonesia comparing a single dose of artemisinin/naphthoquine to a 3-day regimen of dihydroartemisinin/piperaquine came to similar conclusions.^32^ Further studies and the the manufacturing company’s adherence to the WHO ´s standards could result in a new viable ACT recommended by the WHO.

**Fig. 4:**
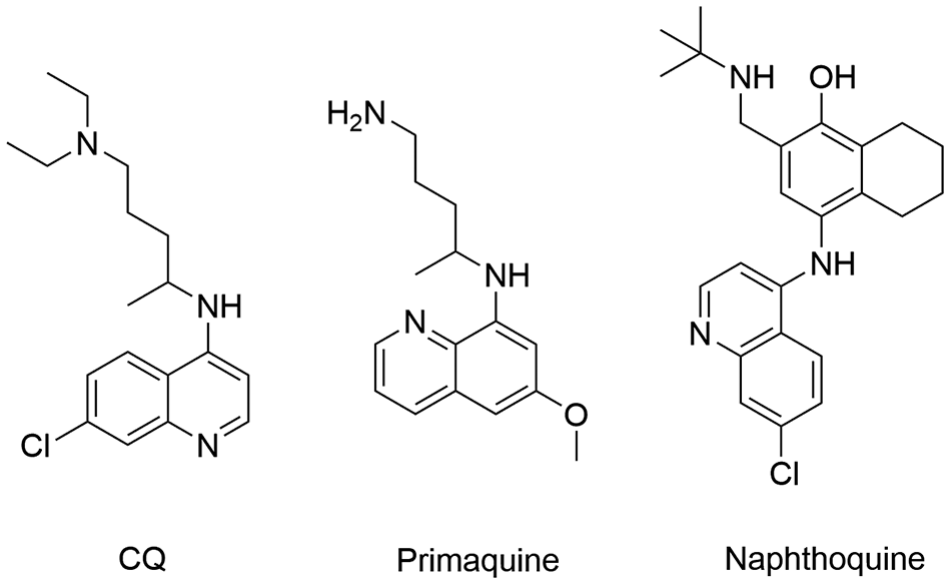
chloroquine, primaquine and naphthoquine chemical structures

Primaquine (Fig. 4) is an 8-aminoquinoline able to kill mature gametocytes of *P. falciparum*, schizonts of all species and it is the only current drug used in chemotherapy, eliminating the latent hypnozoite of *P. vivax* and *P. ovale*, thus providing a radical cure.^33^ In an open trial in Cambodia, the gametocytic efficacy of PQ in a single dose was assessed in association with ACT, in a region with a known ACT resistance. In this report, PQ significantly reduced gametocytemia and efficiently prevented the transmission of the *Plasmodium* agent to mosquitoes, blocking further transmission to new vectors.^34^ Its precise mechanism of action is still unknown, but there is evidence that mitochondrial function is impaired through ubiquinone inhibition, which leads to interference in the respiratory chain.^35^ An excellent review about six decades after primaquine’s discovery, including some of its most important derivatives, was provided by Vale et al.^36^

Chloroquine (CQ) (Fig. 4) is the main representative of the 4-aminoquinoline class, being widely employed as the first-line treatment for uncomplicated *P. falciparum* infection. However, resistance to this drug is widespread and CQ is no longer used for this species. In addition, this drug is applied to uncomplicated malaria caused by chloroquine-sensitive strains of *P. vivax*, *P. ovale* and *P. malariae*.^5^ ^,^ ^24^ The most accepted hypothesis for 4-aminoquinolines’s action mechanism is inhibition of haematin crystallisation. This is the main mechanism of heme detoxification in *Plasmodium* parasites, occurring within the food vacuole, in which the acidic environment aids the chloroquine accumulation, due to its weak alkaline nature.^36^ ^,^ ^37^

Tafenoquine (TQ) (Fig. 5) is another 8-aminoquinoline, which has been assessed in several clinical trials so far. In a phase IIb study, TQ demonstrated a higher efficacy than its precursors for preventing relapse of malaria caused by *P. vivax* (91.9% of TQ versus 77.3% of PQ). In total, thirteen assays were performed to support the efficacy of TQ, with special attention to three randomised, double-blind studies: DETECTIVE Part 1 and Part 2 (NCT01376167) and GATHER. TQ was approved by the FDA in July 2018, and is currently produced by GlaxoSmithKline^®^, under the brand *Krintafel*.^38^ ^,^ ^39^ ^,^ ^40^

Ferroquine (Fig. 5) is a 4-aminoquinoline containing a ferrocene moiety, synthesised in 1994, which demonstrated exceptionally good efficacy against CQ-resistant *P. falciparum* strains whilst possessing low toxicity.^41^ The efficacy of ferroquine monotherapy and the association ferroquine/artesunate against amodiaquine-artesunate (AQAS) was assessed in a phase IIa, open label, clinical trial in Africa (Kenya and Gabon). Both groups exhibited no parasitaemia 28 days after the treatment and showed similar efficacy. However, ferroquine patients presented some adverse reactions, including an alanine aminotransferase increase, alkaline phosphatase and QT interval prolongation (the combination of cardiac depolarisation and repolarisation) all of which are known adverse reactions to 4-aminoquinolines. Thus, future patients treated with ferroquine must have their hepatic and cardiac profiles monitored.^42^ ^,^ ^43^

**Fig. 5:**
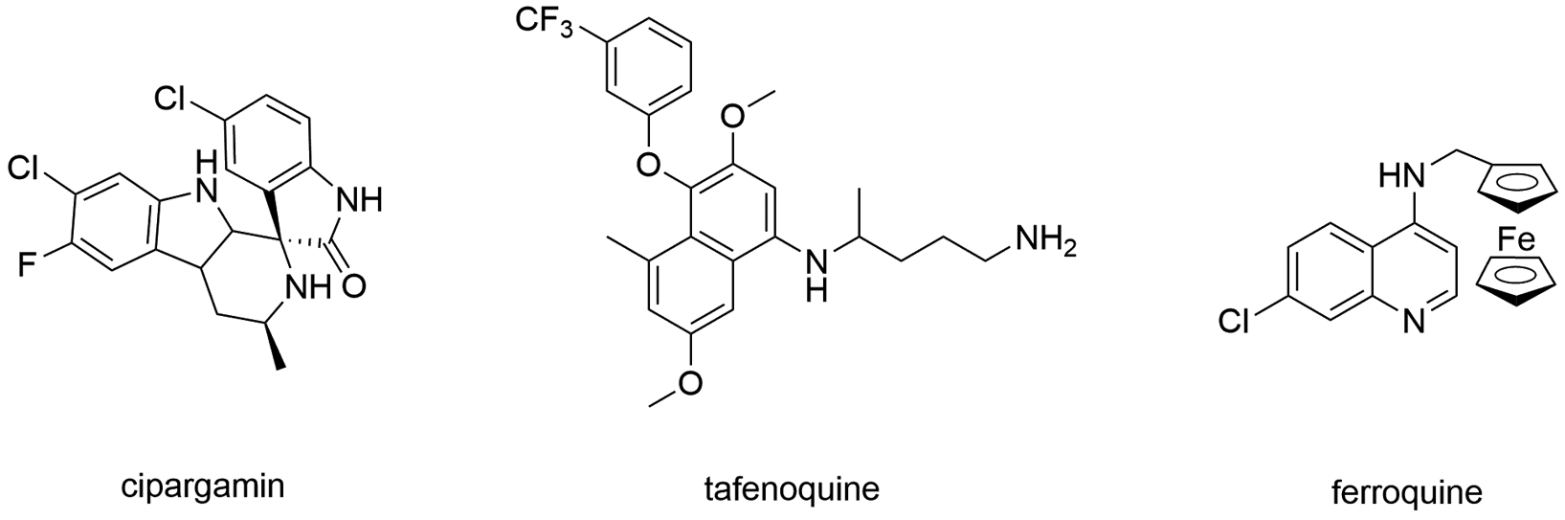
new drugs on malaria chemotherapy.

Cipargamin (Fig. 5 - NITD609, Novartis^®^) is a spiroindolone in Phase II clinical trials. This compound inhibits a new molecular target, PfATP4, present in *P. falciparum* - the first new validated molecular target for malaria after 20-year research. PfATP4 is a Na^+^-ATPase responsible for maintaining a low concentration of cytosolic sodium, which, when inhibited, causes a disturbance of parasitic sodium haemostasis, leading to its death. Cipargaramin derivatives can achieve an IC_50_ lower than 0.2 nM and display activity against oocytes, gametocytes and the asexual stage present in *Plasmodium* parasites blood. Phase I clinical trials used an estimated dose based on the PK/PD 30 mg model, demonstrating no major adverse events, with minor gastrointestinal and genitourinary events when high doses were administered. The drug showed a dose dependent effect, reaching a plateau of activity in doses around 30 mg. The completion of the Phase II clinical trial is due in 2020.^39^ ^,^ ^44^ ^,^ ^45^

Fig. 6 shows the sites of action of the classical and new antimalarial drugs.

**Fig. 6:**
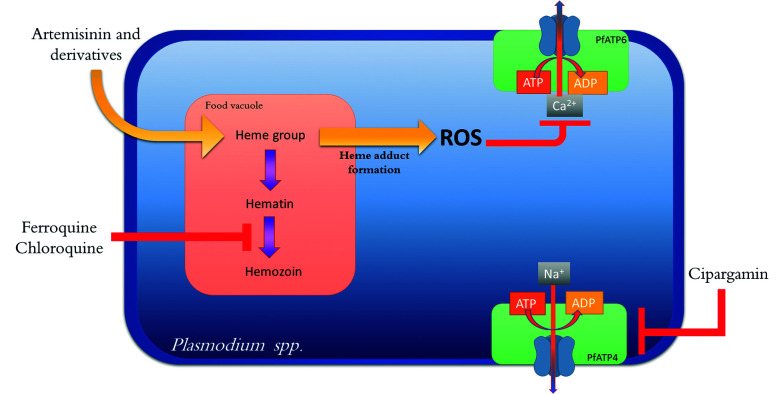
sites of action of some classical and new antimalarial drugs.

Repurposing, also called repositioning, reprofiling or re-tasking^46^ is an approach that has been highly valued for NTD. This is completely understandable, as it encompasses the new use for an approved or investigational drug for other disease that is different from its primary indication. This reduces the risk and the cost when compared to new drugs, since the bioactive compound has already passed through clinical testing. Therefore, it is also a powerful tool for discovering new antimalarial drugs.

A study performed by Pazhayam and co-workers (2018) screened 226 FDA-approved drugs against *P. falciparum* and *P. berghei*. Applying 2.5 µM IC_50_ as threshold. A total of 18 compounds presented significant efficacy, ranging from 2.2 mM to 0.29 mM. Four of them are Over The Counter (OTC) drugs (clemastine fumarate, loperamide hydrochloride, omeprazole and esomeprazole magnesium - Fig. 7), a desirable characteristic for endemic disease in developing countries, where these are mostly found.^47^

Fosmidomycin (Fig. 7) is an antibiotic derived from phosphonic acid that was shown to interfere with the nonmevalonate pathway of *P. falciparum*. Its combination with clindamycin has already been trialed and has presented initial success for malaria treatment, however, a more recent trial reported unfavorable results for this combination.^9^ ^,^ ^29^ Fosmidomycin was also trialed in combination with primaquine in Gabon and showed promising results. The study was designed as a proof-of-concept and evaluated the efficacy, tolerability and safety of the combination therapy and thus did not include a control population.^48^ Further work must be done to properly assess the viability of this combination.

**Fig. 7:**
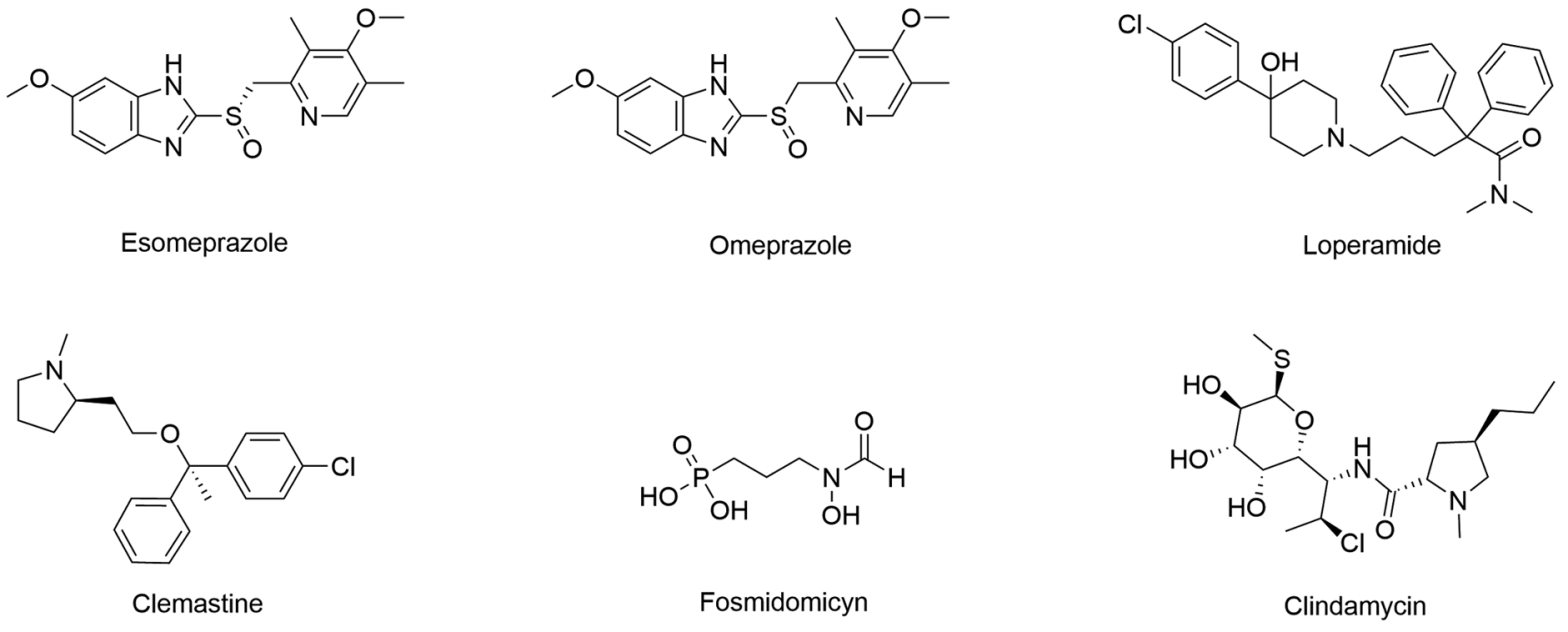
repurposing drugs with activity on *Plasmodium falciparum* and *P. berghei*.

Differently than other repurpose drugs that intend to kill *Plasmodium* parasites, ivermectin is being considered for mass administration to control malaria vectors.^49^ ^,^ ^50^ ^,^ ^51^ The drug is an endectocide with human and veterinary applications,^49^ ^,^ ^50^ but it is also reported that it is able to reduce the lifespan of mosquitoes who consumed blood containing ivermectin from people treated with the drug.^49^ ^,^ ^50^ ^,^ ^52^ This, in turn, reduces malaria transmission by decreasing the chance of the mosquito spreading the disease.^49^ ^,^ ^51^ The insecticide effect mechanism is based on the binding of the drug to glutamate-gated chloride channels.^50^

There are at least 50 new bioactive compounds previously quoted and novel drugs currently underway for malaria treatment, in lead optimisation, preclinical and clinical evaluations.^24^ ^,^ ^47^ It is noticeable that the global efforts to find new therapeutic options are working satisfactorily, with significant new bioactive molecules being synthesised and a considerable number of new academia-industry corporations and university partnerships engaged in this purpose.

Tuberculosis chemotherapy

*Current status* - Although there are established treatment regimens (Table I), TB is still a huge challenge to health systems around the world, due to MDR-TB and XDR-TB strains. MDR-TB is defined by WHO as a form of TB which is resistant to isoniazid and rifampicine. In addition to these two drugs, XDR-TB strains are also resistant to at least one fluoroquinolone and one injectable second-line drug, such as aminoglycosides.^53^ Reports have been showing that XDR-TB is teaming up with MDR-TB in some regions, raising attention and concerns about future outbreaks. Taking this fact into consideration, the WHO established that Direct Observation Treatment Short Course (DOTS) should be applied to new patients as a method for minimising treatment negligence, and research efforts must be employed to avoid the spread of resistance.^54^ ^,^ ^55^

**TABLE I:** Tuberculosis treatment.^51^

Phase	Medicines	Duration (months)
Intensive	HRZ+ E or SM	Two
Continuation	HR	Four-seven

Currently, there are two lines of TB treatment standardised by the WHO. The first one employs four of the following: isoniazid (H), rifampicin (R), pyrazinamide (Z) , ethambutol (E) (Table II and Fig. 8), preferably under DOTS.^56^ ^,^ ^57^ Fluoroquinolones such as levofloxacin and moxifloxacin may also be employed in case of resistant strains, despite not being approved by the FDA. Rifabutine can substitute R in human immunodeficiency viruses (HIV) positive patients, as it causes a lot of negative drug-drug interactions with antirretroviral agents. The second is useful for MDR/XDR-TB, including several drugs from different classes, being the most used: cacycloserine, ethionamide, para-amino-salicylic acid and the aminoglycosides kanamycin, amikacin, capreomycin and streptomycin. These latter drugs are not recommended as a first line of treatment, since they may not be as effective as the former ones and may induce adverse reactions.^54^ ^,^ ^58^ For more details on toxicity, adverse reactions and other treatments regimens of TB, there are some excellent reviews available.^58^

**TABLE II:**
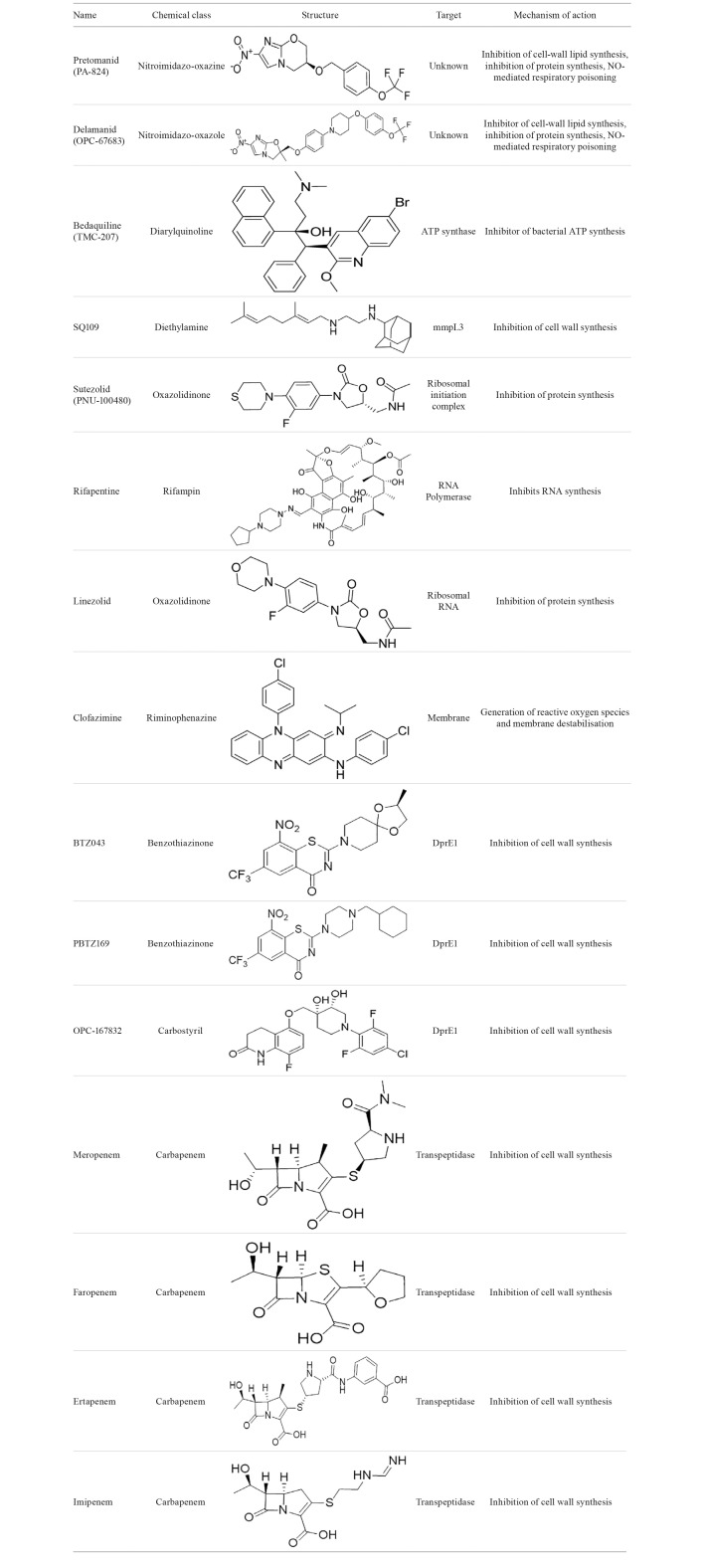
Identification, molecular structure and mechanism of the novel anti-tuberculosis drugs.^67^

**Fig. 8:**
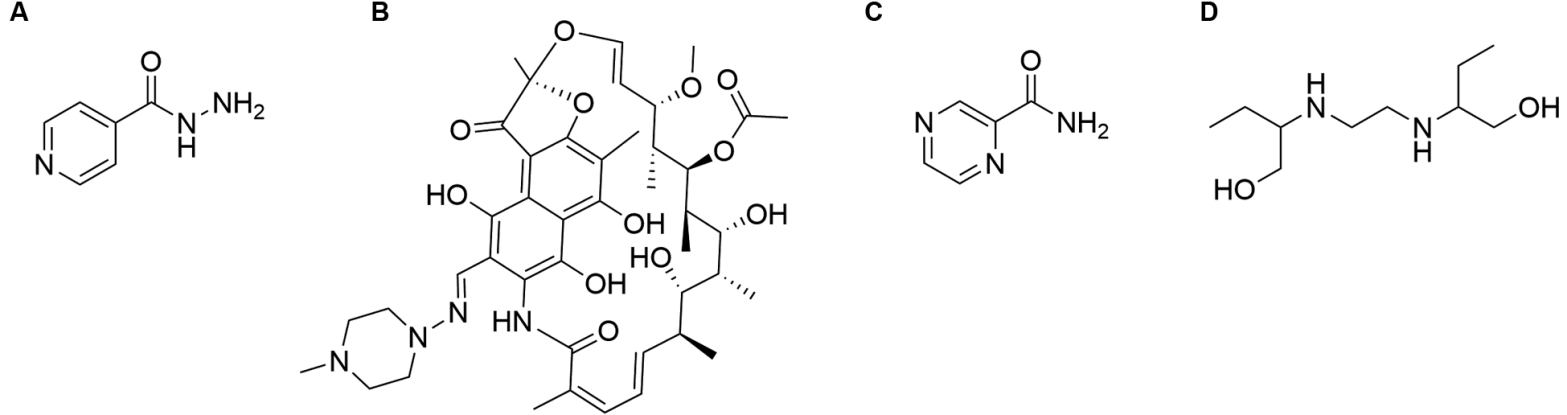
main tuberculostatic drugs: (A) isoniazid (H); (B) rifampicin (R); (C) pyrazinamide (Z); (D) ethambutol (E).

*Advances in the research of new tuberculostatic agents* - The introduction of rifampicin and its first use in 1966 marked the last drug released for a long period of time, as the therapeutic regimen was effective enough to treat infected patients.^59^ However, the emergence of a resistant strain changed this view and since 2000, new efforts arose, leading to the discovery and development of novel bioactive molecules, which are shown on Table II.

Bedaquiline (TMC-207) (Table II) is a diarylquinoline, inhibiting the proton pump of mycobacterial ATP synthase, leading to ATP depletion and an imbalance in pH homeostasis.^60^ Bedaquiline, when added to an optimal treatment regimen for MDR-TB and XDR-TB, provided higher and earlier sputum culture conversion when compared to the control, improving the efficacy of the standard treatment and presenting a good safety profile for administration to HIV infected patients, with modest side effects or drug-drug interactions with antiretroviral therapy.^61^ ^,^ ^62^ Additionally, human mitochondrial ATP synthase displayed more than 20,000-fold lower selectivity to TMC-207, when compared to mycobacterial ATP synthase, preventing toxicity in mammalian cells.^62^ ^,^ ^63^ ^,^ ^64^

SQ109 is a diethylamine (Table II) related to ethambutol, inhibiting cell wall synthesis. Bacilli exposed to SQ109 showed immediate inhibition of trehalose dimycolate, failing to attach mycolates to the arabinogalactan. SQ109 demonstrated better results *in vitro* and *in vivo* when compared to the standard treatment protocol.^65^ ^,^ ^66^

Linezolid (Table II) is an oxazolidinone, which inhibits bacterial protein synthesis through its binding to rRNA and prevents elongation of peptide chains. Hence, linezolid has been repurposed as a tuberculostatic agent in recent clinical trials and has shown significant advances. The drug has proven to be efficient in fluoroquinolone resistant MDR or XDR, improving sputum culture conversion and significantly raising the success rates of this treatment.^68^ ^,^ ^69^ Linezolid presented high level of mutant prevention against *M. tuberculosis*, comparatively to some fluoroquinolones.^70^ ^,^ ^71^ Even though linezolid is a promising treatment for fluoroquinolone-resistant MDR, its use must be carefully monitored, as this drug presents several adverse effects, the most alarming being peripheral and optic neuropathy, which should be assessed in the current phase III clinical trials (CT: NCT02333799, NCT02754765).^72^ ^,^ ^73^ ^,^ ^74^

Sutezolid (PNU-100480) (Table II) is an oxazolidinone derivative from linezolid, which affects bacteria by inhibiting protein synthesis, and has been found to be more potent than linezolid.^75^ In the first clinical trial, sutezolid was shown to be safe with detectable bactericidal effects on sputum and blood.^76^

Rifapentine (Table II) is a rifampicin analog with a similar mechanism of action and has recently been recommended as a drug for treatment of Latent Tuberculosis infection (LTBI) in combined therapy with isoniazid. In comparison to the standard regimen therapy for LTBI and to 3-month isoniazid-rifapentine regimen, the latter exhibits a similar efficacy of isoniazid monotherapy for six or nine months, presenting a low frequency of adverse effects and a higher completion rate.^77^ ^,^ ^78^ ^,^ ^79^

Pretomanid (PA-824) (Table II) is a bicyclic imidazole able to destroy both replicant and non-replicating bacilli through different mechanisms. According to the authors, the inhibition of mycolic acids leads to cell wall disruption (isoniazid-like), which is the death-inducing effect of PA-824 against replicating/active bacteria, while its anaerobic killing activity is related to its NO releasing potential, causing respiratory poisoning in the microorganism.^80^ ^,^ ^81^ Additionaly, pretomanid was shown to cause the accumulation of metabolites such as ribose-5-phosphate, fructose-6-phosphate and glyceraldehyde-3-phosphate, which leads to accumulation of methylglyoxal, a reactive aldehyde that can interact with proteins and DNA, generating toxicity and causing cell arrest. It is a prodrug activated by a deazaflavin dependent nitroreductase (Rv3547).^82^ The non-replicating cells have proven to be particularly hard to eradicate and, therefore, responsible for long-term infection, while also being related to the latent tuberculosis form, which is estimated to affect around one-third of the entire human population. Pretomanid was approved by the FDA in early-2019.^83^

Delamanid (OPC-67683) (Table II) is another nitro-dihydro-imidazooxazole derivative, which similarly to pretomanid, is also a prodrug that inhibits the biosynthesis of mycolic acids, protein synthesis and induces respiratory poisoning through NO generation.^67^ ^,^ ^84^ ^,^ ^85^ Combination therapy studies of delamanid with rifampicin and pyrazinamide demonstrated a faster sterilisation of lung tissue than the standard regimen containing HRZE.^85^ Delamanid presents an outstanding low minimum inhibitory concentration (MIC) against TB and MDR-TB strains, in addition to not being mutagenic.^86^ In an optimal background regimen, it can significantly improve cure or treatment completion rates, and also sharply reduce death rates, although OPC-67683 is associated with prolongation of the QT interval.^87^ ^,^ ^88^ In addition, delamanid does not interact with major antiretroviral drugs, which makes its use optimal for HIV positive patients.^89^

Clofazimine (Table II) is a lipophilic riminophenazine dye employed for the treatment of leprosy. This compound was originally synthesised as a dye and was repurposed as a tuberculostatic agent in 1954, though inconsistent results caused it to be repositioned to treat leprosy later. Nowadays, clofazimine activity against MDR-TB and XDR-TB is being reconsidered and it has been suggested by the WHO as a drug for the treatment of resistant strains of *M. tuberculosis*. Clofazimine acts as a prodrug in *M. tuberculosis* by reduction through the NADH dehydrogenase (NADH2) enzyme, releasing reactive oxygen species upon reoxidation by O2.^90^ Clofazimine has shown better efficiency than control treatments in some studies, although there is a concern over the side effect of reddish-brown skin discoloration, observed in up to 94% of the treated patients.^91^ These findings suggest that clofazimine enhances the activity of other tuberculostatic drugs, such as pyrazinamide, fluoroquinolones, amikacin and *para*‐aminosalicylic acid.^92^

In the last few years, carbapenems such as meropenem and faropenem (Table II) have surfaced as options for MDR and XDR-TB. Although initially discredited as a treatment for TB due to inefficacy caused by mycobacterial beta-lactamases, some studies have demonstrated its efficiency when associated with beta-lactamase inhibitors such as clavulanate.^93^ ^,^ ^94^ The mechanism of action is thus, analogous to the mechanism on other microorganisms, through binding of mycobacterial transpeptidase and preventing the crosslinking of aminoacids on the cell wall, leading to inhibition of its synthesis. Meropenem has been evaluated through in vivo studies to show activity against *M. tuberculosis*, and retrospective studies on patients have shown that meropenem-clavulanate have added value to multidrug treatments, elevating the sputum conversion rate to levels as high as 87%.^93^ ^,^ ^95^ ^,^ ^96^ Faropenem has also showed activity against mycobacterial transpeptidase *in vivo*, presenting a 6- to 22-fold more efficient inhibition than meropenem.^97^ Some clinical trials are currently evaluating the efficacy of meropenem and faropenem combined to amoxicillin and clavulanate, as well as the PK/PD of ertapenem in patients with TB (CT: NCT01730664).^98^ Nowadays, even though the efficacy and overall safety of carbapenems for TB are still being evaluated and are under clinical trials, meropenem, imipenem and ertapenem can already be prescribed in cases of XDR-TB (CT: NCT01730664, NCT03174184, NCT03237182, NCT03625739NCT03237182).^99^ ^,^ ^100^ ^,^ ^101^ ^,^ ^102^ ^,^ ^103^

BTZ043, a benzothiazinone, and OPC-167832, a carbostyril derivative, are members of a novel class of drugs (Table II) for TB that target the enzyme decaprenylphosphoryl-β-d-ribose 2’-oxidase (DprE1).^104^ ^,^ ^105^ DprE1 is a flavoenzyme that plays a vital role in the production of arabinan for the cell wall biosynthesis, leading to its disruption when inhibited, highlighting its potential as a drug target.^106^ BZT043 is the lead compound representant of the benzothiazinones and one of the most potent of the series, presenting a MIC of 1 ng/mL and good synergy with other tuberculostatic drugs in *in vitro* assays, and is now under phase II clinical trials.^105^ ^,^ ^107^ ^,^ ^108^ PBTZ169 is another benzothiazinone (Table II) that proved itself very potent in *in vitro* assays, with a MIC of 0.2 ng/mL, and is currently under phase II clinical trials.^109^ ^,^ ^110^ Similarly, OPC-1677832 (Table II) presented excellent results *in vitro*, achieving a MIC ranging from 0.001 to 0.000024 μg/mL on 40 different strains of *M. tuberculosis*, including MDR and XDR strains, and is now currently under phase II clinical trials.^104^ ^,^ ^111^

Currently, there are more than fifteen clinical trials in Phase II or III, evaluating the efficacy of new tuberculostatic drugs in combination therapy regimens, alone or in association with another standard drug.^55^ Several compounds are in Phase II clinical trials such as delpazolid, nitazoxanide, SQ109 , Q203, carbapenems, benzothiazinones and OPC-1677832.^112^ Some of these compounds explored new targets, such as SQ109, which targets mmpL3 and inhibits the donation of mycolic acid to the cell wall, and BTZ043, which inhibits DprE1, both promoting the cell wall disruption. Even if these analogs fail in their clinical trials, they may cast a light and serve as lead compounds for optimisation and later discovery of new tuberculostatic drugs.

Fig. 9 shows, schematically, the sites of action of most of the compounds shown at Table II.

**Fig. 9:**
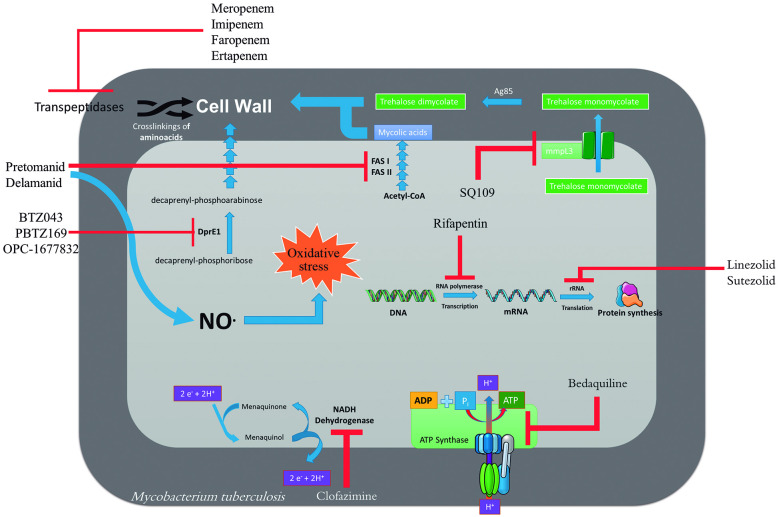
sites of action of tuberculosis drugs/bioactive compounds.

The efficacy of a novel formulation for a low dose rifampicin regimen (200 mg compared to the standard 450 mg), piperine (10 mg) and isoniazid (300 mg), named risorine, was assessed in a Phase III clinical trial in comparison to the standard treatment regimen, conducted in India. The results showed a slightly higher sputum conversion rate and cure rate in the risorine group, when compared to the control. Higher blood levels of rifampicin were achieved in risorine, in relation to the standard regimen, despite the lower dose administered, hence an improvement on the safety profile, with a lower rate of adverse effects.^113^ Lansoprazole, a proton pump inhibitor (PPI), has revealed a high *in vitro* activity against *M. tuberculosis*, while other PPIs such as omeprazole and pantoprazole showed no activity. A cohort study was performed in the UK to assess the incidence of TB on users of PPIs, comparing lansoprazole users to omeprazole or pantoprazole users. Results demonstrated that lansoprazole users presented a considerably lower TB incidence, when compared to omeprazole or pantoprazole users.^114^ The cohort study highlights the importance of lansoprazole studies in treatment for TB, due to its established safety profile, wide availability and low cost.

The global distribution of tuberculosis is still undetermined, with the appearance of MDR and XDR-TB strains gradually spreading more widely. However, considering the history of tuberculosis treatment research, there has been a substantially greater advance in the discovery of biologically active compounds, leads and repurposed drugs in the last 10 years, when compared to the previous 40. In order to achieve global control of this epidemic, some changes are necessary, such as decrease in treatment duration, targeting MDR or XDR and simplifying treatment by lowering dosing frequency. Most of the above-mentioned compounds in this review achieved in some degree these aims.^115^

Drug repurposing has also been stimulated for TB, especially to overcome the severe problem of multi-resistant and extensively drug resistant mycobacteria.^116^

There are some significant programs and institutions, such as the TB Alliance and the WHO, who focus on developing treatments, the political will and on providing orientation to countries with heavy burdens of tuberculosis. Considering these efforts, it is possible that within the next decades, this disease could be brought under control.

*Researches for antituberculosis and antimalarial drugs from protein kinase inhibitors* - Protein kinases play as key controllers of signal transduction, being responsible for regulating essential cellular processes, such as growth, development and replication.^117^ ^,^ ^118^ ^,^ ^119^ Therefore, human kinases inhibitors have been extensively investigated as therapeutic agents for several diseases, as cancer, inflammatory, and cardiovascular illnesses.^119^ There are large libraries of protein kinase inhibitors, which have been searched in designing of potential antimicrobial drugs against tuberculosis and malaria. However, the development of kinase inhibitors is still a major challenge, due to the lack of knowledge of the role of these proteins in infections. Another relevant point is that most kinase inhibitors compete with ATP and thus, they can inhibit human kinases, presenting limited selectivity.^120^ ^,^ ^121^ ^,^ ^122^ On the other hand, there are significant differences between the ATP binding sites from pathogenic protein kinases to their human homologues. These changes provide substantial differences regarding the selectivity, allowing the designing of new drugs.^122^

The emergence of drug-resistant strains highlights the need of new therapeutic approaches, thereby kinase inhibitors should gain attention as new therapeutic choices against tuberculosis and malaria.^123^ There are host kinases able to act in the immune response against infection and mycobacterial or plasmodial kinases. Herein, we report the enzymes from the pathogen employed as a target for drug design.

Protein kinases participate in phosphorylation processes involved in host-pathogen interaction, being classified into three main groups: serine/threonine protein kinase (STPK), tyrosine protein kinase (TPK) and two-component regulatory system (2CRS) consisting of histidine kinase and response regulator. The 2CRS system responsible for protein phosphorylation from prokaryotes occurs only on His and Asp residues.^123^

*Tuberculosis* - *M. tuberculosis* protein kinases have shown to be critical targets for mycobacterial survival and proliferation.^123^ Differently from other pathogens, *M. tuberculosis* presents in its kinoma 11 STPKs number similar to 2CRSs, playing important roles for its survival, pathogenesis and virulence. These STPKs are named PknA to PknL.^124^ PknA and PknB are two *M. tuberculosis* enzymes widely investigated, and they are essential for growth and regulation of cell wall biosynthesis and cell division.^125^ ^,^ ^126^ PknE, PknG and PknH seem to act in the pathogenesis of tuberculosis.^127^ ^,^ ^128^ ^,^ ^129^ In this context, STPK inhibitors have been intensively evaluated and some have shown promising antimycobacterial activity. A high-throughput screening identified potential inhibitors against purified PknB STPK. However, the most active compounds did not induce death by *M. tuberculosis* in cell culture.^130^ Probably, the thick and “waxy” cell wall prevents the inhibitors from reaching the site of action. The findings showed higher activity of these compounds in cells treated with wall breakdown reagents. Therefore, these compounds can undergo molecular modifications, in order to improve their permeability in mycobacteria.^128^ Tetrahydrobenzothiophene derivatives were identified as PknG inhibitors and the compound AX20017 (Fig. 10) displayed promising anti-Tb activity in whole-cells assays, being able to decrease the survival of *M. tuberculosis* inside the macrophages.^131^

2CRSs from *M. tuberculosis* are essential for the mycobacterial growth, *Mt*rA and *Mt*rB perform in the regulation of mycobacterial metabolism and adaptation to environmental changes.^132^ Banerjee and co-workers (2016) carried out a virtual screening for identifying *Mt*rA inhibitors and they found eight potential molecules. Biological assays revealed that the compounds 2IT4O (2-iminothiazolidine-4-one - Fig. 10) and OTABA (oxo-1, 3-thiazolidin-2-ylidene amino benzoic acid - Fig. 10) inhibited *Mt*rA. Furthermore, these compounds decreased the mycobacterial growth *in vitro* with IC_50_ of 9 μM and 34 μM, respectively. Finally, 2IT4O displayed IC_50_ value (3 μM) lower in macrophages, implying it can also act in other pathways.^133^

**Fig. 10:**
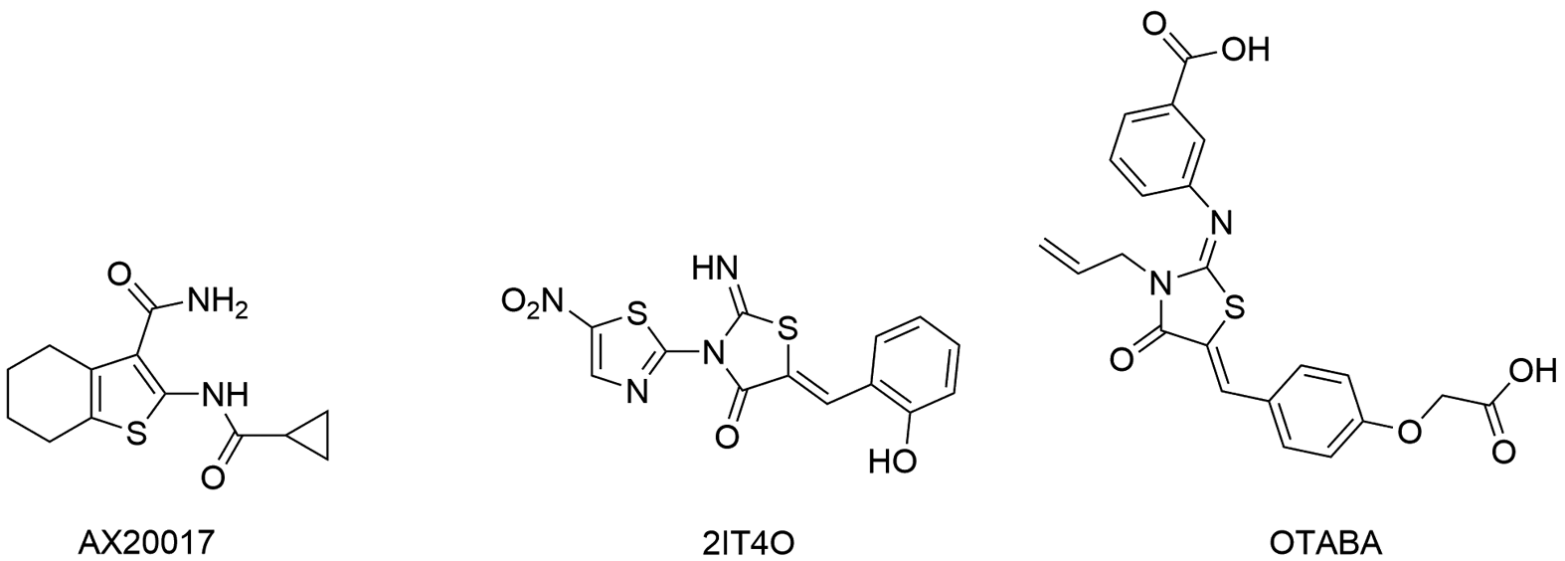
kinase inhibitors active in *Mycobacterium tuberculosis*.

*Malaria* - In antimalarial therapy, there are no protein kinase inhibitor drugs. However, these enzymes present an essential role in the host and in the parasitic life cycle. In this context, protein kinase inhibitors are a promising field for designing of antimalarial agents. *P. falciparum* protein and lipid kinases participate in the main signaling pathways at diverse steps of its life cycle.^134^ *P. falciparum* kinome encodes 86 to 99 protein kinases and a small set of lipid kinases, though the function of most of them is still unknown. The most advanced studies involve calcium-dependent protein kinases (*Pf*CDPKs), Protein kinase 7 (*Pf*PK7), cyclin-dependent kinases (*Pf*MRK), cGMP-Dependent Protein Kinase (*Pf*PKG), Phosphoinositide lipid kinases (PIKs).^122^ ^,^ ^123^

CDPKs from *Plasmodium* (*Pf*CDPK) belongs to the STPK family and are composed of seven members named as *Pf*CDPK1 to *Pf*CDPK7. They are one of the most attractive targets for designing of new antimalarial agents due to the critical role in the life cycle of *Plasmodium* and absence of their homologous in the human, which may result in higher selectivity against parasite and lower toxicity to the host.^135^ ^,^ ^136^

*Pf*CDPK1 phosphorylates important proteins of the parasitic motor complex involved in the invasion of host cells, especially in erythrocytes, being considered a remarkable target for antimalarial therapy.^137^ ^,^ ^138^ ^,^ ^139^ Several compounds showed inhibitory activity against *Pf*CDPK1. Imidazopyridazine derivatives, 2, 6, 9-trisubstituted purines, and bisindolocarbazole K252a (a staurosporine analogue) showed to be active in protein kinase and/or *P. falciparum* survival assays in host cells.^137^ ^,^ ^138^ ^,^ ^140^ ^,^ ^141^ ^,^ ^142^ In murine model, the imidazopyridazine compound (Fig. 11) significantly reduced the survival of *P. berghei* ranging from 46% to 51%, when administered orally once daily in doses of 50 mg/kg for four days.^141^ ^,^ ^142^

**Fig. 11:**
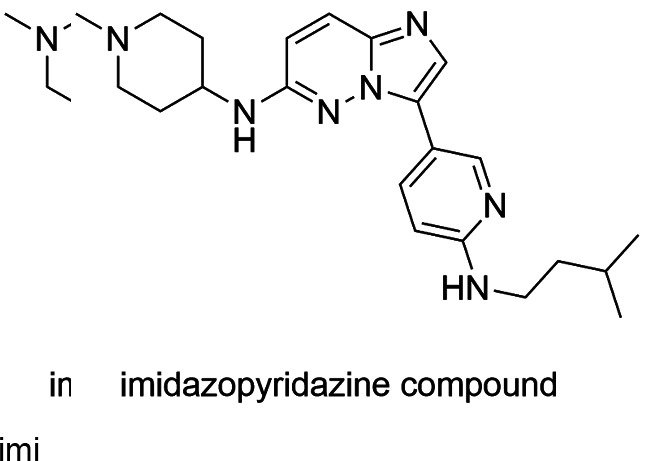
imidazopyridazine derivatives, an inhibitor of *Pf*CDPK1.

*Pf*CDPK4 participates in the sexual cycle of *Plasmodium* in *Anopheles*.^143^ In *P. berghei*, *Pb*CDPK4 regulates gamete formation mediated by xanthurenic acid and parasite transmission.^144^ In a *Pb*CDPK4 knockout assay, male gametocytes were not able to mature into fertile male gametes in the mosquito gut. Thereby, this kinase prevents the parasite transmission, blocking the parasitic ex-flagellation in the gametogenesis process inside the vector.^145^ CDPK4 contains a serine residue in the gatekeeper position on the ATP binding pocket. This residue provides a larger ATP-binding site than all mammalian homologues, increasing selectivity for kinase from *P. falciparum*. In this context, pyrazolopyrimidine and imidazopyrazine compounds inhibited from *Pf*CDPK4, among them Bumped Kinase inhibitors, as the derivative of BKI-1 (Fig. 12) presented IC_50_ of 4 nM *Pf*CDPK4 and also it was validated in a mouse model. This analogue did not show toxicity and suppressed exflaggelation for up to 14 h at doses of 50 mg/kg administered intraperitoneally in *P. berghei* infected mice, revealing good efficacy and pharmacokinetic properties.^144^ ^,^ ^145^ ^,^ ^146^

**Fig. 12:**
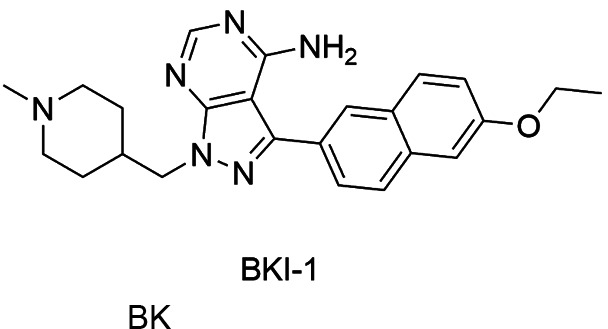
bumped kinase inhibitor (BKI-1) able to *Pf*CDPK4 inhibit.

*Pf*PK7 (*P. falciparum* protein kinase 7) plays at different stages of the parasite life cycle, both in vector and in the human host. It was found that the suppression of the *Pf*PK7 gene attenuates the parasite asexual growth in erythrocytes, thus *Pf*PK7 acts a crucial role in the transmission.^147^ Several *Pf*PK7 inhibitors have been described, such as compounds K510, K109, K497, imidazopyridazine, himenialdisine and staurosporine.^148^ ^,^ ^149^ Merck and co-workers (2008) reported that the compounds K510, K109 and K497 (Fig. 13) suppressed asexual growth of *P. falciparum* in blood-stage cells.^149^ Bouloc et al. described the inhibitory activity of imidazopyridazines against *Pf*PK7 and *Plasmodium* in *in vivo* assays.^148^ It is worth highlighting that imidazopyridazines also are potent inhibitors of *Pf*CDPK1, thereby they can play through two different pathways.^140^ ^,^ ^141^ ^,^ ^142^ ^,^ ^150^

**Fig. 13:**
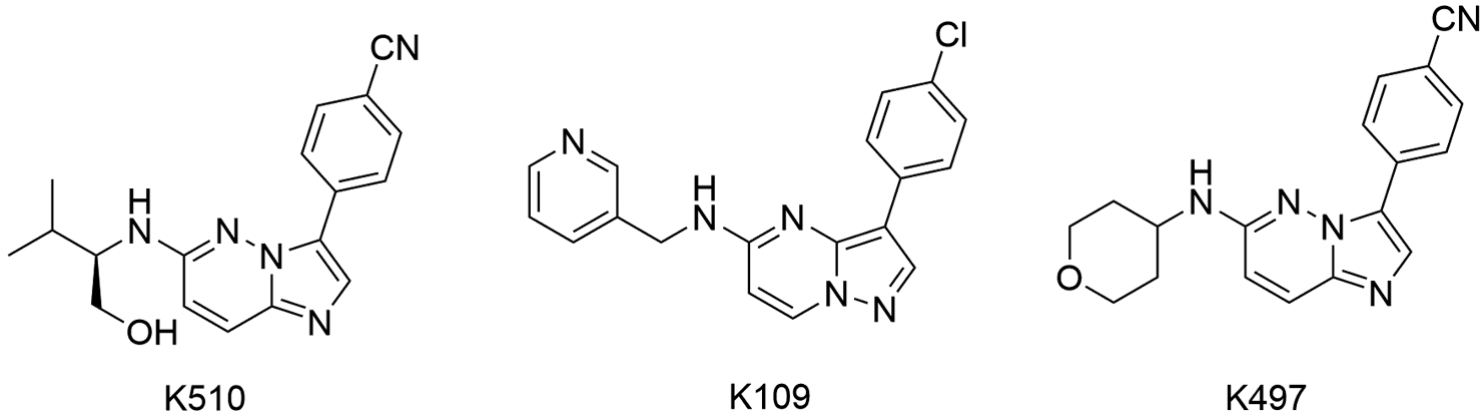
inhibitors of *Pf*PK7, compounds K510, K109 and K497.

*Pf*PKG (*P. falciparum* cGMP-Dependent Protein Kinase) is a protein involved in multiple steps of *Plasmodium* life cycle, being essential for the replication process in the host blood-stage.^151^ Its inhibition resulted in the release of mature non-invasive schizonts^152^ ^,^ ^153^ ^,^ ^154^ and participates also in gametogenesis,^155^ mobility^156^ and the late development of the liver-stage.^157^ Imidazopyridines showed potent inhibitory activity against *Pf*PKG important to the sexual stage of *P. falciparum*, blocking the gametocyte transmission to the vector.^158^ The most potent compounds 13 and 14 (Fig. 14) exhibited IC_50_ values of 130 and 160 pM, respectively, against the wildtype enzyme, and 2 and 102 nM against the wildtype *Pf*3D7 strains. Furthermore, compound 14 displayed good results regarding cytotoxicity, moderate metabolic stability *in vitro* and high selectivity against a panel of 80 human kinases. The oral administration of analogue 14, twice daily with doses at 100 mg/kg during four days in mice infected with *P. falciparum* led on the reduction of parasitaemia to undetectable levels.^158^

**Fig. 14:**
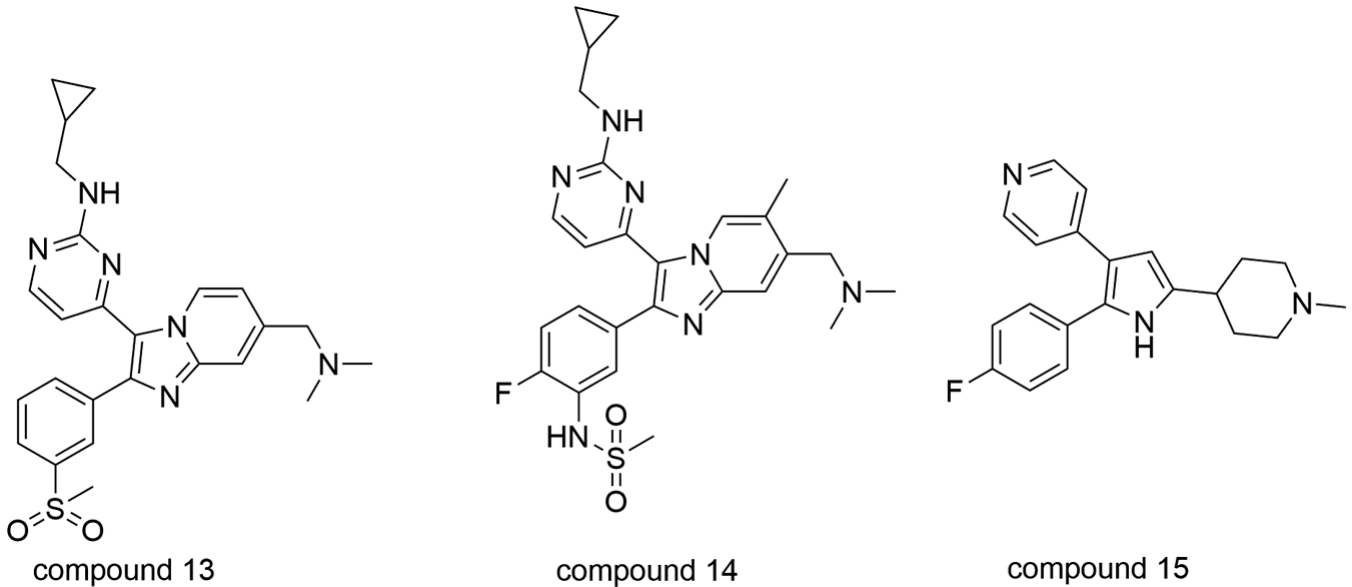
inhibitors of PKG, compounds 13, 14 and 15.

From a series similar to the imidazopyridine composed of 2,3-diaryl-pyrrole derivatives, compound 15 (Fig. 14) was found and exhibited IC_50_ of 3.5 nM against recombinant *Pf*PKG, similar to the native strain.^159^ *In vitro* assay against chloroquine-sensitive strain *Pf*NF54 and the chloroquine-resistant strain *Pf*Dd2, the compound 15 displayed IC_50_ values of 0.49 and 1.3 μM, respectively. On the other hand, in mice infected with *P. berghei*, compound 15 was not able to eliminate parasites at intraperitoneal doses of 50 mg/kg twice daily for eight days. In HepG2 cell culture, compound 15 decreased the infection of *P. berghei* by sporozoites.^139^ In addition, *in vitro*, compound 15 decreased the number of parasites in the liver-stage below the detection limit, inhibiting host cells invasion with an IC_50_ below 1 μM. However, sporozoites without *Pb*PKG remained sensitive to compound 15, indicating that it affects other targets than *Pb*PKG. In mouse infected with *P. yoelii*, strain with higher infectivity of the sporozoite than *P. berghei*, before the infection was administered a single intraperitoneal dose of 50 mg/kg, which resulted in reduction of hepatic parasitic load by 1000 times. Thereby, three doses of 50 mg/kg were administered, the first 15 minutes before the infection and, second and third doses 6 and 12 h after infection, all mice were free of parasites in the blood-stage during the three week of the experiment.^160^

The *Pf*MRK protein plays an essential role in DNA replication and *Plasmodium* transcriptional control.^161^ ^,^ ^162^ ^)^ From a series of quinolinones, the compound 16 (Fig. 15) was identified as a *Pf*MRK inhibitor with an IC_50_ of 18 μM, but it was not active against D6 strains of *P. falciparum*.^161^ ^,^ ^163^ Research performed by the Walter Reed Army Institute of Research reported the oxindole derivative 17 (Fig. 15) as *Pf*MRK inhibitor (IC_50_ = 1.4 μM) and it showed higher selectivity than mammalian kinase (CDK1 - IC_50_ = 29 μM). However, in sensitive *Pf*D6 strains, the compound 17 showed moderate antiplasmodial activity, which was attributed to the its low permeability.^164^

**Fig. 15:**
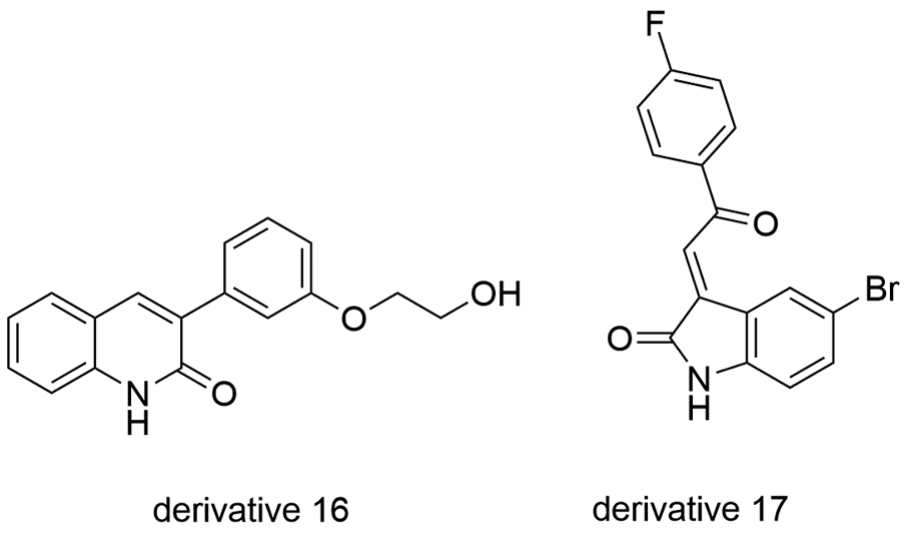
quinolinone and oxindole derivatives, promising inhibitor of *Pf*MRK.

Phosphoinositide lipid kinases (PIKs) generate phosphorylated phosphatidylinositol derivatives, which are crucial for different cellular functions, such as messenger signaling, cell membrane remodeling and vesicular trafficking.^165^ ^,^ ^166^ The most searched PIKs in *Plasmodium* species are phosphoinositide 3-kinase (PI3K) and phosphatidylinositol 4-kinase (PI4K), both are essential for the survival of *P. falciparum*. PI3K acts on the growth of the parasite and was recently reported as one of the targets of dihydroartemisinin, in which dihydroartemisinin demonstrated to be a potent *Pf*PI3K inhibitor in the nanomolar range.^167^

Two research groups are working on PI4K inhibitors, they are the Genomics Institute of the Novartis Research Foundation and the Novartis Institute for Tropical Diseases. They found antiplasmodial activity for series of imidazopyridines, pyrazines, and pyridazines.^168^ ^,^ ^169^ SAR studies were developed and KAI407 (Fig. 16) showed significant activity against *P. falciparum*, *P. yoelii* and *P. cynomolgi*. However, KAI407 exhibited poor physico-chemical properties. In this context, molecular modifications were carried out in the core, which was changed to imidazopyrazine group, resulting in KDU691 compound less lipophilic (Fig. 16). *In vitro*, this derivative was active among *Plasmodium* species, particularly *P. cynomolgi*. KDU691 showed to be effective against clinically resistant isolates of all classes of antimalarials. In addition, this compound was active *in vitro* against the development of the hepatic-stage of *P. yoelii* and against liver resident hypnozoites grown *in vitro* from *P. cynomolgi*, suggesting promising activity against *P. vivax*, responsible for the malaria recurrence. Finally, KDU691 exhibited favorable pharmacokinetic properties, by oral administration of 20 mg/kg prevented the colonisation of mice by *P. berghei*, potential as a prophylactic agent.^168^ ^,^ ^169^

**Fig. 16:**
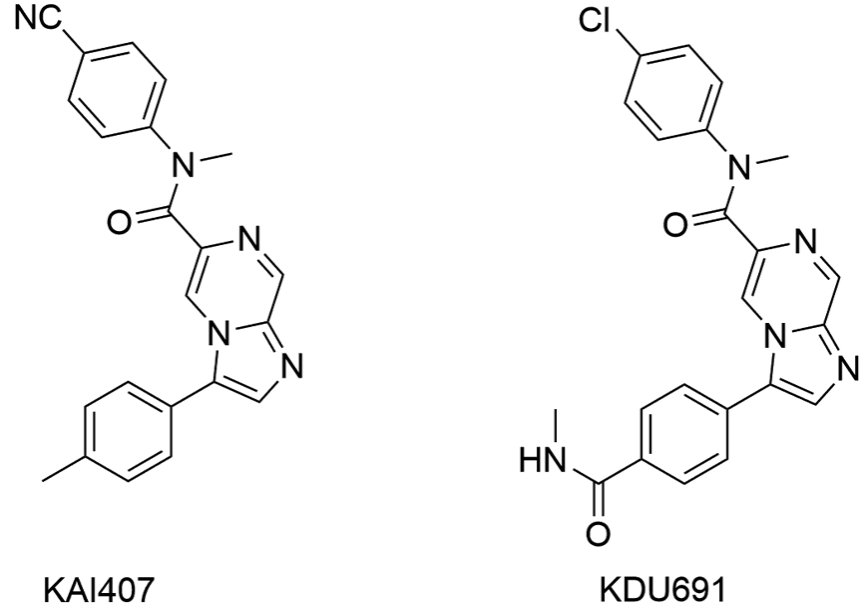
imidazopyrazine compounds potential PI4K inhibitors.

Other class of PI4K inhibitor, aminopyridine and pyrazine compounds were found in a collaboration between the University of Cape Town Drug Discovery and Development Centre and Medicines for Malaria Venture (MMV).^170^ Through molecular modifications, MMV048 (Fig. 17) was obtained and exhibited high activity against sensitive *Plasmodium*, drug-resistant strains, besides the favorable pharmacokinetic properties. MMV048 inhibited *P. vivax* (*Pv*) PI4K with IC_50_ of 3.4 nM,^171^ beyond the close correlation between the enzyme inhibition and total cell activity. The substitution of sulfone by a piperazine amide resulted to UCT943 (Fig. 17), compound with better solubility and greater antiplasmodial activity.^172^ Both compounds displayed to be highly effective *in vivo* models of *Plasmodium* infections, for example, MMV048 presented ED_90_ values of 0.80 mg/kg in mouses infected with *P. berghei*. MMV048 showed prophylactic activity, preventing infection in a monkey model infected with *P. cynomolgi* (2 mg/kg before infection). In addition, its pharmacokinetic properties were evaluated in mice, rat, dog and monkey models. From these assays, a single dose ranging 80 to 100 mg for humans was established. MMV048 progressed from preclinical development to Phase 1 clinical trials. Currently, it is in Phase 2a, thus MMV048 may be the first *Plasmodium* kinase inhibitor to reach therapy.^173^

**Fig. 17:**
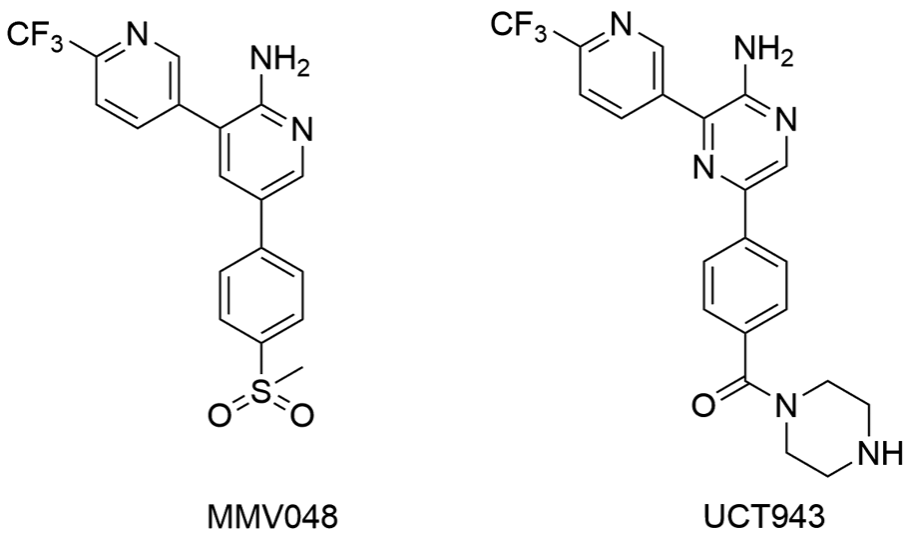
PI4K inhibitors, aminopyridines compounds, MMV048 is in Phase 2 on clinical trials.

Concluding remarks

Although malaria and tuberculosis are not considered neglected tropical diseases by the WHO,^174^ this topic is yet polemic. Drug resistance is a challenge that must be faced for both diseases. Continuous interest must be maintained, as they affect mainly poor, or neglected, people in the world, and are responsible for a high level of mortality in these populations and put at risk many countries, including developed ones.^1^ ^,^ ^6^ ^,^ ^175^ ^,^ ^176^ The intensification and the ease of people’s migration are modifying the spread of many diseases, including those ones. Even considering the higher financial support for malaria and tuberculosis than for other diseases that are considered neglected, the amount invested is still lower than the estimated need.^1^ ^,^ ^6^

Fortunately, many groups in academia, sometimes with the partnership of pharmaceutical industries - consortia maintained by DNDi and MMV are good examples - keep the search for new and better drug candidates alive, primarily for drug resistant diseases. In this sense, this literature review presents advances in the design and discovery of new and promising molecules, some of them already in the clinical phase. It is worth noting that the repurposing approach has been explored, as this is much stimulated due to the advantages it presents.
